# Construction of a Live-Attenuated Vaccine Strain of *Yersinia pestis* EV76-B-SHUΔ*pla* and Evaluation of Its Protection Efficacy in a Mouse Model by Aerosolized Intratracheal Inoculation

**DOI:** 10.3389/fcimb.2020.00473

**Published:** 2020-09-08

**Authors:** Junxia Feng, Yingying Deng, Mengjiao Fu, Xueyuan Hu, Wenbo Luo, Zhiyu Lu, Lupeng Dai, Huiying Yang, Xiaodong Zhao, Zongmin Du, Bohai Wen, Lingxiao Jiang, Dongsheng Zhou, Jun Jiao, Xiaolu Xiong

**Affiliations:** ^1^State Key Laboratory of Pathogen and Biosecurity, Beijing Institute of Microbiology and Epidemiology, Beijing, China; ^2^Capital Institute of Pediatrics, Beijing, China; ^3^Department of Laboratory Medicine, Zhujiang Hospital, Southern Medical University, Guangzhou, China; ^4^Anhui Medical University, Hefei, China

**Keywords:** *Yersinia pestis*, live-attenuated vaccine, aerosolized intratracheal inoculation, mucosal immune, low residual virulence

## Abstract

Plague, which is caused by *Yersinia pestis*, is one of the most dangerous infectious diseases. No FDA-approved vaccine against plague is available for human use at present. To improve the immune safety of *Y. pestis* EV76 based live attenuated vaccine and to explore the feasibility of aerosolized intratracheal inoculation (i.t.) route for vaccine delivery, a plasminogen activator protease (*pla*) gene deletion mutant of the attenuated *Y. pestis* strain EV76-B-SHU was constructed, and its residual virulence and protective efficacy were evaluated in a mouse model via aerosolized intratracheal inoculation (i.t.) or via subcutaneous injection (s.c.). The residual virulence of EV76-B-SHUΔ*pla* was significantly reduced compared to that of the parental strain EV76-B-SHU following i.t. and s.c. infection. The EV76-B-SHUΔ*pla* induced higher levels of mucosal antibody sIgA in the bronchoalveolar lavage fluid of mice immunized by i.t. but not by s.c.. Moreover, after lethal challenge with *Y. pestis* biovar Microtus strain 201 (avirulent in humans), the protective efficacy and bacterial clearance ability of the EV76-B-SHUΔ*pla*-i.t. group were comparable to those of the EV76-B-SHUΔ*pla*-s.c. and EV76-B-SHU immunized groups. Thus, the EV76-B-SHUΔ*pla* represents an excellent live-attenuated vaccine candidate against pneumonic plague and aerosolized i.t. represents a promising immunization route in mouse model.

## Introduction

Plague is a serious infectious disease that is caused by the Gram-negative bacterium *Yersinia pestis*. The disease is mainly endemic among rodents and can be transmitted to humans via the bite of *Y. pestis*-infected fleas (Reed et al., 1970; Perry and Fetherston, 1997; Stenseth et al., 2008). Plague has brought about 3 pandemics in the past, which led to over 200 million human deaths (Perry and Fetherston, 1997). Plague can come in different disease forms, such as pneumonic, bubonic, or septicemic plague. Pneumonic plague is most dangerous among these 3 disease forms because the pathogen can disseminate through aerosol droplets; the mortality of pneumonic plague approaches 100% if treatment is delayed. The infection rate of *Y. pestis* has risen since 1990s, leading plague to be defined as a re-emerging infectious disease (Williamson, 2001). Moreover, antibiotic-resistant *Y. pestis* strains have already emerged (Guiyoule et al., 2001), and *Y. pestis* can be engineered as a bioweapon (Inglesby et al., 2000). Therefore, vaccination remains the first choice for management of the deadly disease.

A killed whole-cell vaccine and the live-attenuated EV76 vaccine were frequently used to prevent plague in the past. Immunization with the killed whole-cell vaccine can induce short-term protection and requires frequent stimulation to maintain immunity (Wang et al., 2013). Live-attenuated vaccines are able to induce long-lasting humoral and cellular immune responses, making them the preferable vaccine type (Derbise et al., 2012; Rosenzweig and Chopra, 2012). The live-attenuated *Y. pestis* EV76 vaccine strain which is pigmentation locus (*pgm*)-lacking can induce protection against bubonic and pneumonic plague (Smiley, 2008a), and it was used during plague endemic periods throughout the world. However, the *Y. pestis* EV76 live attenuated vaccine caused side effects of varying severity (Meyer, 1970; Russell et al., 1995) and also Δ*pgm* mutant of *Y. pestis* caused fatal infection of an individual with hemochromatosis (Frank et al., 2011; Quenee et al., 2012), indicating that the safety of EV76 live-attenuated vaccine still need to be improved. Thus, it is of great importance to construct EV76 based live attenuated strains with additional mutations that maintain a proper balance between virulence attenuation and immunoprotection.

To improve the safety of *Y. pestis* EV76 based live attenuated vaccine while maintaining its vaccine efficacy, we decide to delete Pla because it is an plasminogen activator protease involved in dissemination of *Y. pestis* into circulation, and is one of the major virulence determinants of this pathogen (Sodeinde et al., 1992; Lahteenmaki et al., 2005; Lathem et al., 2007). It was demonstrated Pla^−^ mutant replicated at the site of infection in mice infected at a subcutaneous injection (s.c.) site, but showed only transient replication at peripheral sites (Welkos et al., 1997), suggesting that mutant might stimulate a vigorous immune response. In addition, the Pla protease does not appear to be an effective immunogen in mice and was not protective in mice (Sample et al., unpublished data). Another previous study demonstrated that a CO92 Pgm^−^Pla^−^ strain was more attenuated than the CO92 Pgm^−^ strain in Swiss Webster mice by s.c. and in monkeys by the aerosol route (Welkos et al., 2002). Taken together, these data indicated that deletion of *pla* gene should not decrease the efficacy of a *Y. pestis* live vaccine while increasing its safety. In this study, we demonstrate that a Δ*pla* mutant of *Y. pestis* EV76-B-SHU is attenuated in a mouse model, and further show that it provides excellent protection against *Y. pestis* i.t. infection in a mouse model.

The lungs have a large surface area, abundant blood flow, and highly permeable epithelium, so vaccines administered via the lung show better bioavailability and more rapid onset time than other alternative routes (Patton et al., 2004; Patton and Byron, 2007; Lee et al., 2012; Kunda et al., 2013). At the same time, such administration can induce both long-lasting systemic and mucosal immune responses against respiratory pathogens (Timothy et al., 2015; Perdomo et al., 2016; Wu et al., 2017; Florido et al., 2018). Therefore, pulmonary vaccine delivery has received a lot of attention in recent studies (Guillon et al., 2012; Lee et al., 2012). Intranasal and intratracheal inoculation are the common methods of pulmonary application. Both of them have been used for delivery of subunit vaccines in mouse model (Eyles et al., 1998, 2000). However, these methods have several limitations: the loss of drug in the nose, throat and upper airways, the un-quantification of the given dose, the requirement for invasive surgery. In this study, we introduced an aerosolized intratracheal inoculation (i.t.) route, which is an improved non-invasive method for delivery of vaccines into the lungs of mouse using a Micro Sprayer (Bivas-Benita et al., 2005). Immunization of EV76-B-SHUΔ*pla* via aerosolized i.t. conferred a level of protection comparable to that elicited by immunization of EV76-B-SHUΔ*pla* via s.c, indicating that aerosolized i.t. is a promising immunization route in mouse model.

## Materials and Methods

### Deletion of the *pla* Gene of *Y. pestis* EV76-B-SHU

The 8-907 bp fragment in the coding sequence of *pla* gene from *Y. pestis* EV76-B-SHU was deleted using the CRISPR-Cas12a-assisted lambda RED recombineering system as described previously (Yan et al., 2017) and the primer Oligo-pla and the primer pair Pla-top and Pla-bottom were used (Table 1). The deletion of the *pla* gene was confirmed by PCR analysis using inner primers (Pla-inner-F and Pla-inner-R) and outer primers (Pla-F and Pla-R) and by western blot analysis with mouse polyclonal antibody against recombinant Pla protein, the expression of F1 was detected by western blot analysis with mouse monoclonal antibody as control.

**Table 1:** The primers used in this study.

**Primer or primer pair**	**Primer sequence (5^**′**^-3^**′**^)**
Oligo-pla	TGTATTTTTCAGAAGCGATATTGCAGACCCGCCGTCACAGTCTTCATTAGACACCCTTAATCTCTCTGCATGAACGAAA
Pla-top	TCGGAATTTACATACAGTAAATATGAGT
Pla-bottom	TCATATTTACTGTATGTAAATTCCGATC
Pla-F	AATTCTGTCAGACGACGAG
Pla-R	CGTTCCATGTCTAATTTGAC
Pla-inner-F	ATTATAACTATTCTGTCCG
Pla-inner-R	ATCTCCGCCAATAGAGACA

### Complementation of *pla* Gene in EV76-B-SHUΔ*pla*

The coding region of the *pla* gene, along with a 300-nucleotide region upstream of *pla* was synthesized and cloned into the pACYC184 plasmid vector, creating the recombinant plasmid pACYC184*Ypla*, then the pACYC184*Ypla* recombinant plasmid was transformed into *Y. pestis* EV76-B-SHUΔ*pla* mutant strain by electroporation. The complementation of *pla* gene was confirmed by PCR analysis using primers Pla-inner-F and Pla-inner-R and by western blot analysis with mouse polyclonal antibody against recombinant Pla protein, the expression of F1 was detected by western blot analysis with mouse monoclonal antibody as control (Figure S1).

### Bacterial Strains and Growth Media

The *Y. pestis* live-attenuated vaccine strain EV76-B-SHU (Δ*pgm* and ΔYPO1165-1172 compared to CO92), EV76-B-SHUΔ*pla*, and the WT biovar Microtus strain 201 which is avirulent in humans were cultivated in Brain Heart Infusion broth (BHI; BD, Voigt Global Distribution Inc., Lawrence, KS). Overnight cultures were inoculated with BHI in 1:20 and cultured at 26°C to a density at 600 nm of ~1.0, then the cultures were inoculated with BHI in 1:100 and continue cultured at 26°C to the same density at 600 nm of ~1.0. After that, the cultures were transferred to 37°C table concentrator for additional 3 h. For growth on a solid surface, *Y. pestis* was grown on 5% sheep blood agar (SBA) plates (Luqiao, Beijing, China) at 26°C for 3 days.

### Safety Assessment of EV76-B-SHUΔ*pla*

Female BALB/c mice (aged 6–8 weeks) were purchased from Vital River Laboratories (Beijing, China). Animal studies were performed in an Animal Biosafety Level-3 (ABSL-3) laboratory and were carried out with the permission of the Institute of Animal Care and Use Committee (IACUC) of the Academy of Military Medical Sciences (AMMS). All efforts were made to minimize animal suffering. The ethical approval number was IACUC of AMMS-13-2017-022.

Cultured bacterial cells were harvested, washed, and diluted in phosphate-buffered saline (PBS) to an optical density of 1.0 at 600 nm (OD_600_). The bacterial number was calculated by spreading dilutions on SBA agar plates. Mice (*n* = 10/per group) were infected by i.t. or s.c. with the EV76-B-SHU or EV76-B-SHUΔ*pla* strain. Each strain was inoculated at 3 different doses (1×10^4^ CFU, 1×10^6^ CFU, 1×10^8^ CFU). Then the mice were monitored for 14 days to assess the survival rate. For i.t. infection, 50 μl of EV76-B-SHU or EV76-B-SHUΔ*pla* was aerosolized and delivered into the lungs of mice by a Micro Sprayer (Huironghe Company, Beijing, China) with the help of laryngoscope (Huironghe Company, Beijing, China) (Feng et al., 2019). For s.c. infection 100 μl of EV76-B-SHU or EV76-B-SHUΔ*pla* was subcutaneously injected into the inner thigh of mouse.

### Animal Immunization and Challenge

Mice (*n* = 28/ per group) were immunized 3 times at 3-week intervals (days 0, 21, and 42) by i.t. or s.c. For i.t., mice were inoculated with 1 × 10^6^ CFU/50 μl of the EV76-B-SHUΔ*pla* or EV76-B-SHU strain at each immunization. For s.c., mice were immunized with 1 × 10^6^ CFU/100 μl of the EV76-B-SHUΔ*pla* or EV76-B-SHU strain at each immunization. The naïve mice served as negative control, which were not immunized. On day 21 after the last immunization, the immunized mice were anesthetized by intraperitoneal injection of pentobarbital sodium and then infected with 1.22 × 10^3^ CFU/50 μl (61 LD_50_) of *Y. pestis* 201 by i.t. Mice were monitored for 14 days to assess mortality. On day 2 and day 14 post-challenge, 3 mice per group were sacrificed to harvest lungs, spleens, and livers; then about 100 mg of organs were obtained, and homogenized in 800 μl PBS; 50 μl of homogenate were diluted serially in PBS and 10 μl dilutions were plated onto SBA plates; plates were incubated at 26°C for 3 days; CFU were counted and bacterial loads were calculated.

### Antibody Responses

Sera and bronchoalveolar lavage fluids (BALF) from 4 mice per group were collected on day 21 after first immunization (day21), day 21 after second immunization (day42), and day 21 after third immunization (day63), respectively. Levels of IgG and secretory IgA (sIgA) were evaluated by enzyme-linked immunosorbent assay (ELISA) to sonicated *Y. pestis* 201. Briefly, 5 μg/ml sonicated *Y. pestis* 201 was coated overnight on 96-well enzyme-linked plates (Nunc, Shanghai, China) in carbonate buffer and blocked with 2% bovine serum albumin (BSA). Serum and BALF samples were 2-fold serially diluted from 1:100 to 1:102,400 and from 1:2 to 1:2,048, respectively. The diluted serum or BALF was added singly to each well, and wells were incubated with antigen at 37°C for 45 min. The plates were washed 5 times with phosphate buffered saline containing 0.1% Tween-20 (PBST) and then incubated with 100 μl of HRP-conjugated sheep anti-mouse IgG (Abcam,) or anti-mouse IgA (Abcam) at 37°C for 45 min. After another 5 washes, the antibody titers were detected by a TMB substrate kit and analyzed with a MULTISKAN MK3 plate reader at 450 nm. Then the titers of specific antibody were calculated as the reciprocal of the lowest sample dilution giving a signal equal to 2 times the background OD values. Background values were obtained from samples collected from the naïve mice.

### T Cell Recall Assay

On day 21 after each immunization, spleens were collected from 3 mice per group. T cells were isolated from the suspension of each spleen and plated into 24-well plates (Corning, Corning, NY) in RPMI 1640 medium (Gibco, Grand Island, NY) containing 10% FCS for 5 × 10^6^ cells per well as previously described (Xiong et al., 2014). Then T cells in each well were stimulated with 20 μg of sonicated *Y. pestis* 201 antigens or 2.5 μg of ConA at 37°C and 5% CO_2_. After 48 h, the culture supernatant of each well was collected for detection of tumor necrosis factor (TNF)-α, interferon (IFN)-γ, interleukin (IL)-2, and IL-4 using Th1/Th2/Th9/Th17/Th22/Treg Cytokine 17-Plex Mouse Panel kits (Thermo, Vienna, Austria).

### Histopathology

The lungs, spleens, and livers from 3 mice per group were collected 21 days after the last immunization and 2 days post-challenge (day 65). Naïve mice which were not immunized and which were challenged by *Y. pestis* 201 on day 63 served as negative controls. Organ tissues were fixed in 4% paraformaldehyde, and the fixed tissues were sliced, mounted on slides, and stained with hematoxylin-eosin (HE). Pathological alterations in the tissue slices were observed by light microscopy. Tissue sections were evaluated blinded by a trained pathologist according to the following scores: 0, no pathological lesions; 1, minimal; 2, mild; 3, moderate; 4, severe. The degree of pathological lesions was related to the distribution and severity of lesions as follows: (I) thickened alveolar walls; (II) edema; (III) tissue parenchymatous lesions, such as congestion and hemorrhage.

### Statistical Analyses

All statistical analyses were performed using SAS statistical software. However, Kaplan–Meier survival estimates were performed using GraphPad Prism. The differences in bacterial load, antibody levels, and cytokine levels between the EV76-B-SHU and EV76-B-SHUΔ*pla* immunization groups were assayed using two-way analysis of variance (ANOVA), followed by least significant difference (LSD) and Tukey's tests. The animal survival rate was analyzed by Kaplan–Meier survival estimates. *P* < 0.05 was considered significantly different for all statistical analyses.

## Results

### Construction of *Y. pestis* EV76-B-SHUΔ*pla* Mutant

**Figure 1:**
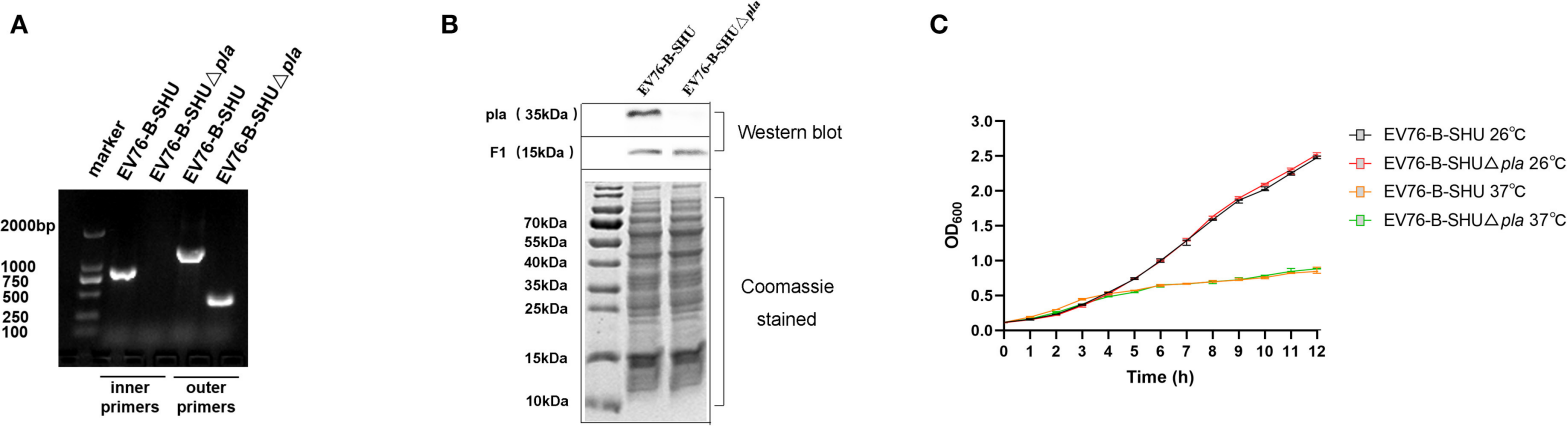
*In-vitro* biological characteristics of EV76-B-SHUΔ*pla*. **(A)** *Pla* gene identification. The *pla* gene of the mutant strain was identified through PCR using inner primers (including Pla-inner-F and Pla-inner-R) and outer primers (including Pla-F and Pla-R). **(B)** Pla and F1 protein expression levels in *Y. pestis* strains. The whole-cell lysates of EV76-B-SHU and EV76-B-SHUΔ*pla* were separated by SDS-PAGE and stained with Coomassie brilliant blue (lower panel), then probed with Pla- specific polyclonal antibody and F1-specific monoclonal antibody, respectively (upper panel). This picture is a composite of the representative result of multiple samples. **(C)** EV76-B-SHUΔ*pla* and EV76-B-SHU bacteria were cultured overnight and then diluted to an OD_600_ of 0.1. The dilutions were cultured in BHI medium at 26°C or 37°C for 12 h. The OD_600_ value was measured at 1-h intervals.

To improve the immune safety of *Y. pestis* EV76 based live attenuated vaccine, the *pla* gene was deleted in the *Y. pestis* live-attenuated vaccine strain EV76-B-SHU. PCR analysis using primers specific to *pla* showed the absence of *pla* in the mutant strain (Figure 1A). Western blot analysis showed that the Pla protein was not detected in mutant cells, although it was detected in the parental strain. The F1 protein levels were similar in mutant and isogenic parental strains, as the corresponding gene was not deleted (Figure 1B). The growth rate of EV76-B-SHU and EV76-B-SHUΔ*pla* at 37°C was slower than that at 26°C (Figure 1C). But the growth rate of the EV76-B-SHUΔ*pla* was not different from that of EV76-B-SHU at 26°C or 37°C, indicating that the deletion of the *pla* gene has no influence on the growth of bacteria *in vitro*.

### Safety Assessment of EV76-B-SHUΔ*pla* Following i.t. or s.c. Inoculation

**Figure 2:**
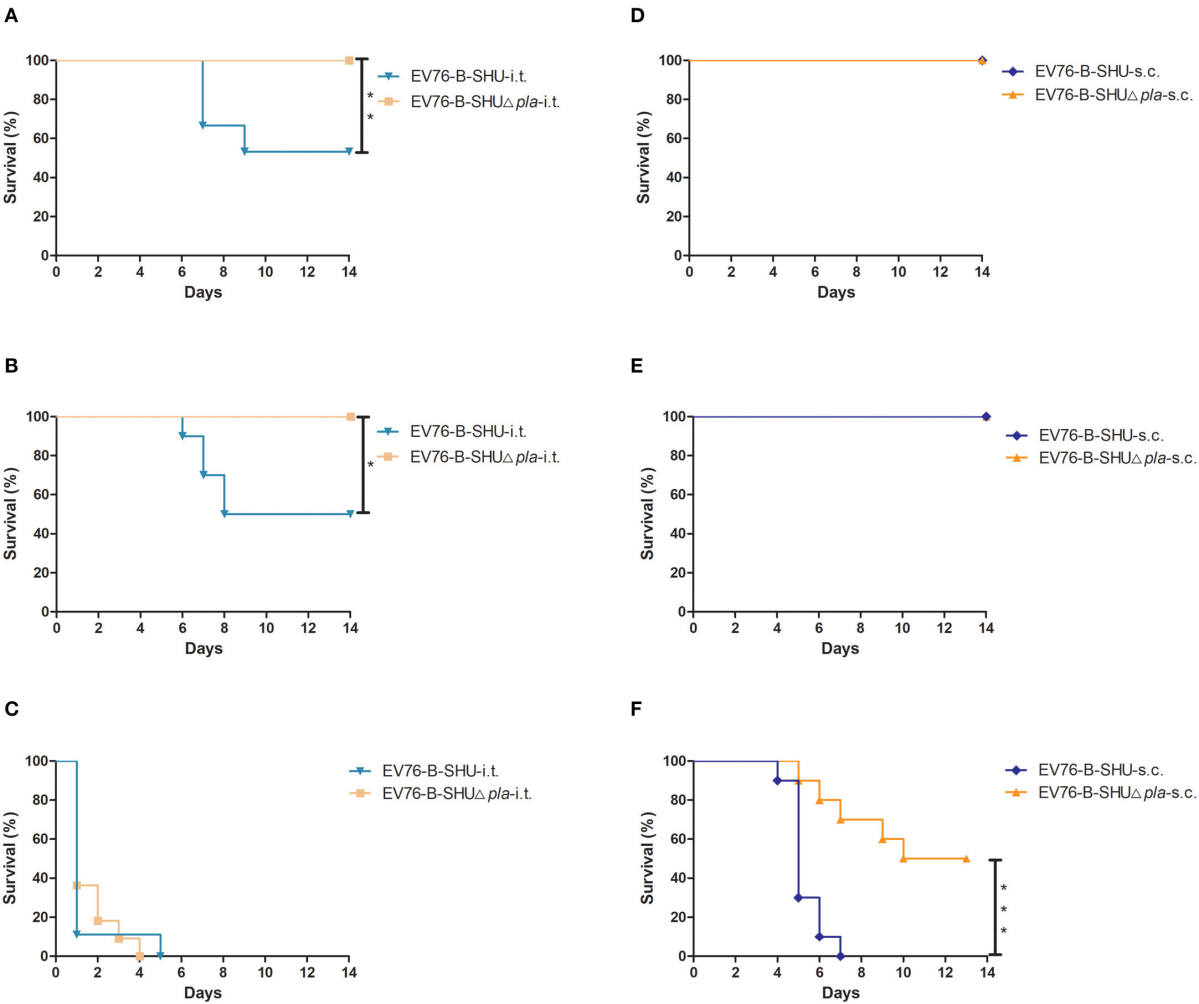
Safety analysis of EV76-B-SHU and EV76-B-SHUΔ*pla* following i.t. or s.c. inoculation. Mice (*n* = 10/per group) were infected by **(A–C)** i.t. or **(D–F)** s.c. with a dose of **(A,D)** 10^4^ CFU, **(B,E)** 10^6^ CFU, or **(C,F)** 10^8^ CFU of EV76-B-SHU or EV76-B-SHUΔ*pla* strain. Then mice were monitored for 14 days. Survival data were analyzed by using Kaplan–Meier survival estimates, and *P* < 0.05 were considered statistically significant. Data are from one representative experiment of two independent experimental determinations.

The safety profile of the EV76-B-SHUΔ*pla* was examined by infecting mice via i.t. or s.c. with doses of 10^4^ CFU, 10^6^ CFU, or 10^8^ CFU of mutant strain. As shown in Figure 2, for i.t., a survival rate of 100% was observed for mice infected with 10^4^ CFU and 10^6^ CFU of EV76-B-SHUΔ*pla* strain, while survival rates of mice infected with 10^4^ CFU (*P* < 0.01, Figure 2A) and 10^6^ CFU (*P* < 0.05, Figure 2B) of EV76-B-SHU were about 50%. The survival rates of mice infected by i.t. with 10^8^ CFU of EV76-B-SHU or EV76-B-SHUΔ*pla* were both 0% (Figure 2C). For s.c., the survival rates of all 10^4^ CFU- and 10^6^ CFU-infected mice were 100% (Figures 2D,E); following inoculation at a dose of 10^8^ CFU, a significantly higher survival rate was detected in the EV76-B-SHUΔ*pla* group than in the EV76-B-SHU group (*P* < 0.001, Figure 2F). Complementation of *pla* with a pACYC184 plasmid restored the virulence of EV76-B-SHUΔ*pla* strain to a significant level via i.t. route (*P* < 0.001, Figure S2) or s.c. route (*P* < 0.05, Figure S2), indicating that the *pla* gene deletion was responsible for the virulence attenuation of EV76-B-SHUΔ*pla* strain in mice.

### Evaluation of Humoral and Lung Mucosal Immune Response by ELISA After Immunization of Mice With EV76-B-SHUΔ*pla* or EV76-B-SHU via i.t. or s.c. Route

**Figure 3:**
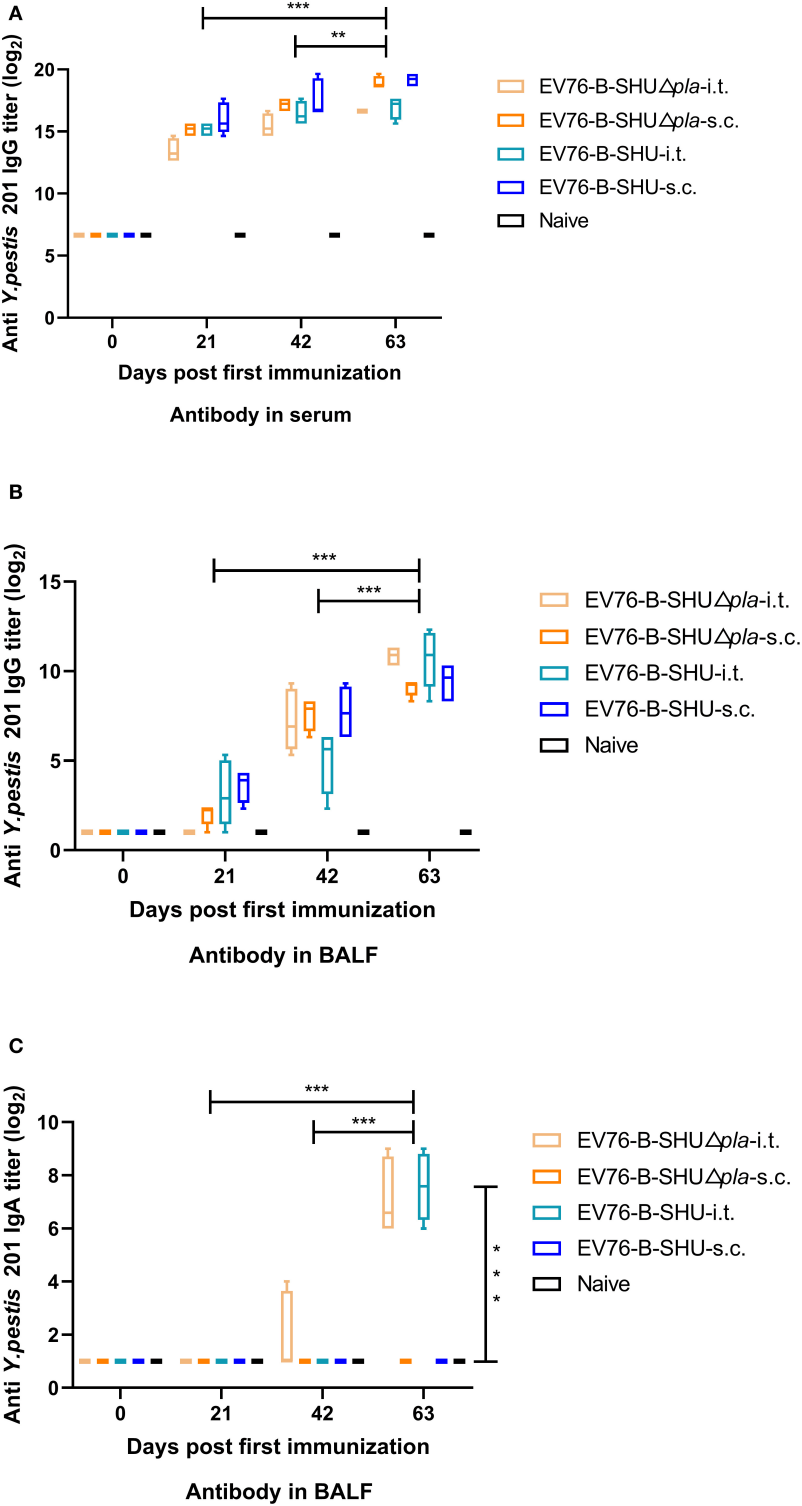
Humoral and mucosal antibody responses in mice elicited by EV76-B-SHU or EV76-B-SHUΔ*pla* via i.t. or s.c. immunization. Mice were immunized by 3 inoculations with 10^6^ CFU EV76-B-SHUΔ*pla* or EV76-B-SHU vaccine (on days 0, 21, and 42) by i.t. or s.c. Serum and BALF were collected from 4 mice from each group on day 21 after each immunization. The titers of IgG and sIgA to sonicated *Y. pestis* 201 were examined by ELISA. **(A)** The reciprocal serum titers of IgG to sonicated *Y. pestis* 201. **(B)** The reciprocal BALF titers of IgG to sonicated *Y. pestis* 201. **(C)** The reciprocal BALF titers of sIgA to sonicated *Y. pestis* 201. The experiments were performed twice independently with similar results. Data are expressed as the mean ± SD (*n* = 4) that collected from one representative experiment. *P < 0.05; **P < 0.01; ***P < 0.001.

Mice were immunized 3 times at 21 days intervals with EV76-B-SHUΔ*pla* or EV76-B-SHU by i.t. or s.c., and serum and BALFs were collected on day 21 after first immunization (day21), day 21 after second immunization (day42), and day 21 after third immunization (day63), respectively. The serum IgG titers and the BALF IgG and sIgA levels to sonicated *Y. pestis* 201 in mouse were measured by ELISA. We found that immunization with the EV76-B-SHUΔ*pla* or EV76-B-SHU by i.t. or s.c. induced significantly higher levels of IgG to sonicated *Y. pestis* 201 in serum on day 63 than on days 21 and 42 after primary immunization. The specific IgG levels in serum of the EV76-B-SHUΔ*pla*-i.t. group were not significantly different from those of the EV76-B-SHU-i.t. group at any time point (*P* > 0.05, Figure 3A).

The levels of IgG specific to sonicated *Y. pestis* 201 in BALF of the EV76-B-SHUΔ*pla*-i.t. and EV76-B-SHU-i.t. groups were significantly higher on day 63 than on days 21 and 42 after primary immunization. (*P* < 0.001, Figure 3B). However, the levels of IgG specific to sonicated *Y. pestis* 201 in BALF of the EV76-B-SHUΔ*pla*-i.t. group were not significantly different from those of the EV76-B-SHU-i.t. group at any time point (*P* > 0.05, Figure 3B). Likewise, the levels of specific sIgA in BALF were also measured. The titers of specific sIgA in the BALF of all groups were low on days 21 and 42 after primary immunization. But the titers of specific sIgA in the BALF of EV76-B-SHUΔ*pla*-i.t. and EV76-B-SHU-i.t. groups increased significantly on day 63 after primary immunization. The levels of specific sIgA in the BALF of EV76-B-SHUΔ*pla*-i.t. and EV76-B-SHU-i.t. groups were significantly higher than those in the EV76-B-SHUΔ*pla*-s.c. and EV76-B-SHU-s.c. groups on day 63 after primary immunization (*P* < 0.001, Figure 3C).

### Evaluation of Specific Cellular Immune Response After Immunization of Mice With EV76-B-SHUΔ*pla* or EV76-B-SHU via i.t. or s.c. Route

**Figure 4:**
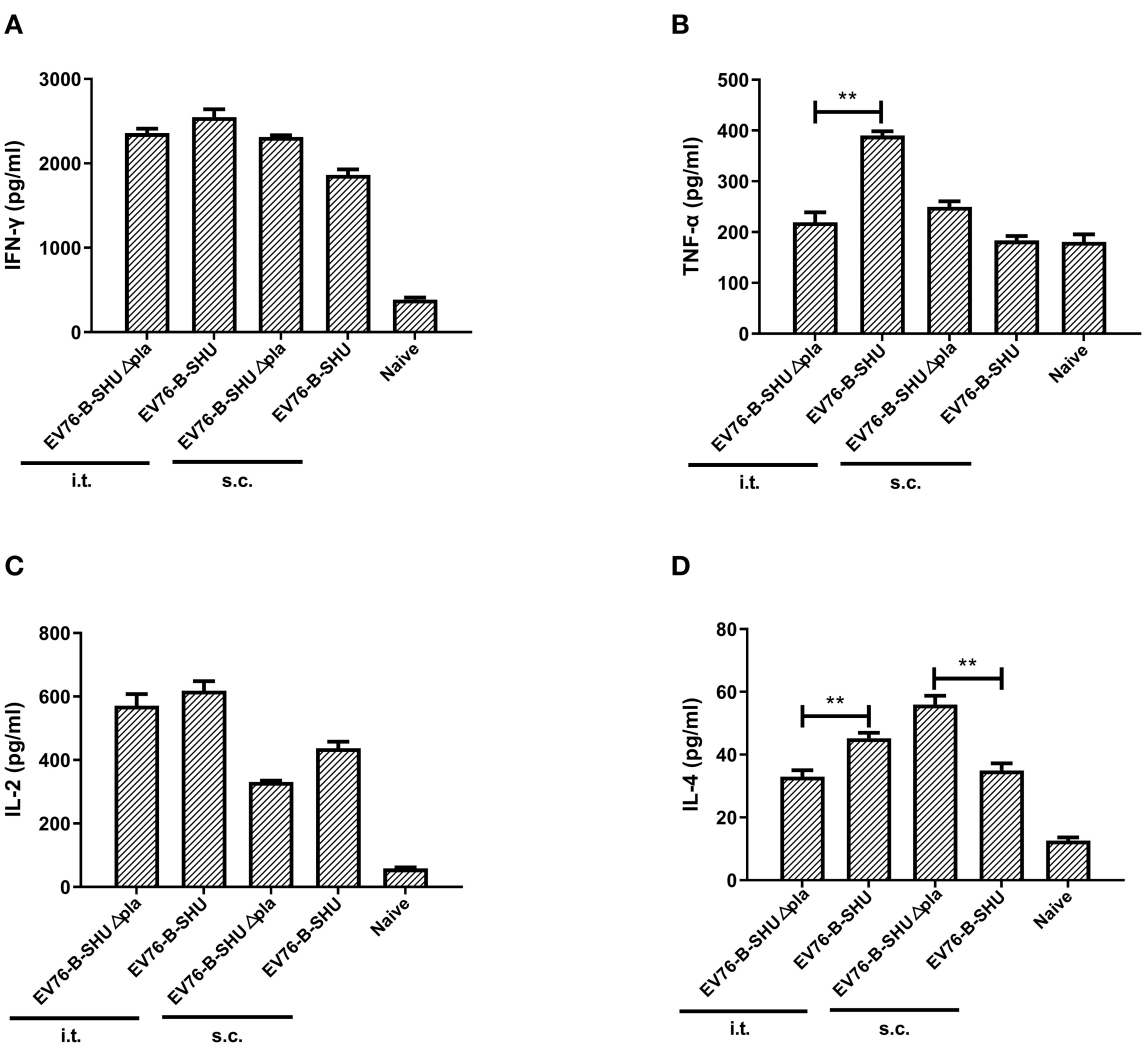
T cell cytokine level after immunization with EV76-B-SHU or EV76-B-SHUΔ*pla* via i.t. or s.c. Mice were immunized by i.t. or s.c. with a dose of 10^6^ CFU of either EV76-B-SHU or EV76-B-SHUΔ*pla*. On day 21 after the first immunization, T cells were isolated from the spleens of 3 mice in each group. The isolated T cells were cocultured with APCs and stimulated with sonicated *Y. pestis* 201. 48 h after culturing, cells were centrifuged, the supernatant was collected, and levels of the cytokines IFN-γ, TNF-α, IL-2, and IL-4 were measured. **(A)** IFN-γ levels. **(B)** TNF-α levels. **(C)** IL-2 levels. **(D)** IL-4 levels. The experiments were performed twice independently with similar results. Data are expressed as the mean ± SD (*n* = 3) that collected from one representative experiment. **P < 0.01.

To investigate the specific primary T cell responses, mice were immunized by i.t. or s.c. with a dose of 10^6^ CFU of either EV76-B-SHUΔ*pla* or EV76-B-SHU. On day 21 after the first immunization, splenic T cells isolated from immunized mice were stimulated with sonicated *Y. pestis* 201 *ex vivo*, and the specific cytokine responses were determined by Luminex assay. The secretion levels of IFN-γ and IL-2 in EV76-B-SHUΔ*pla* immunized groups were not significantly different from that of EV76-B-SHU immunized groups. However, the secretion levels of TNF-α and IL-4 in EV76-B-SHUΔ*pla*-i.t. group was significantly different from that of EV76-B-SHU-i.t. group, and the secretion level of IL-4 in EV76-B-SHUΔ*pla*-s.c. group was significantly different from that of EV76-B-SHU-s.c. group (Figure 4).

### Evaluation of the Protective Effect Provided by i.t. or s.c. Immunization With EV76-B-SHUΔ*pla* or EV76-B-SHU

**Figure 5:**
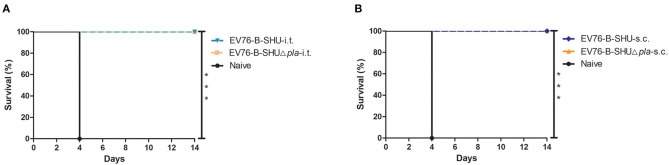
Survival analysis of mice immunized with EV76-B-SHU or EV76-B-SHUΔ*pla* after exposure to *Y. pestis* 201. Mice were immunized with 3 inoculations (10^6^ CFU/dose) of EV76-B-SHUΔ*pla* or EV76-B-SHU strain on days 0, 21, and 42 via i.t. or s.c. Nonimmunized mice served as negative controls. On day 21 after the last vaccination, 10 mice from each group were randomly selected and challenged with 61 LD_50_ of *Y. pestis* 201 via i.t. Mice were monitored for 14 days after challenge. **(A)** Survival analysis of mice immunized with EV76-B-SHUΔ*pla* or EV76-B-SHU by i.t. **(B)** Survival analysis of mice immunized with EV76-B-SHUΔ*pla* or EV76-B-SHU by s.c. Data were analyzed using Kaplan–Meier survival estimates. Vaccination groups were significantly different from the control group (***P < 0.001). Data are from one representative experiment of two independent experimental determinations.

To evaluate the protection efficacy of EV76-B-SHUΔ*pla* and EV76-B-SHU, mice were vaccinated 3 times by i.t. or s.c. with 10^6^ CFU EV76-B-SHUΔ*pla* or EV76-B-SHU. On day 21 after the last immunization, mice were challenged i.t. with a lethal dose of 1.22 × 10^3^ CFU (61 LD_50_) of *Y. pestis* 201. As shown in Figure 5, both i.t. and s.c. immunization with EV76-B-SHUΔ*pla* or EV76-B-SHU provided 100% protection against infection with *Y. pestis* 201. In contrast, all naïve mice succumbed to the challenge.

### Bacterial Enumeration of Mouse Organs After i.t. Challenge

**Figure 6:**
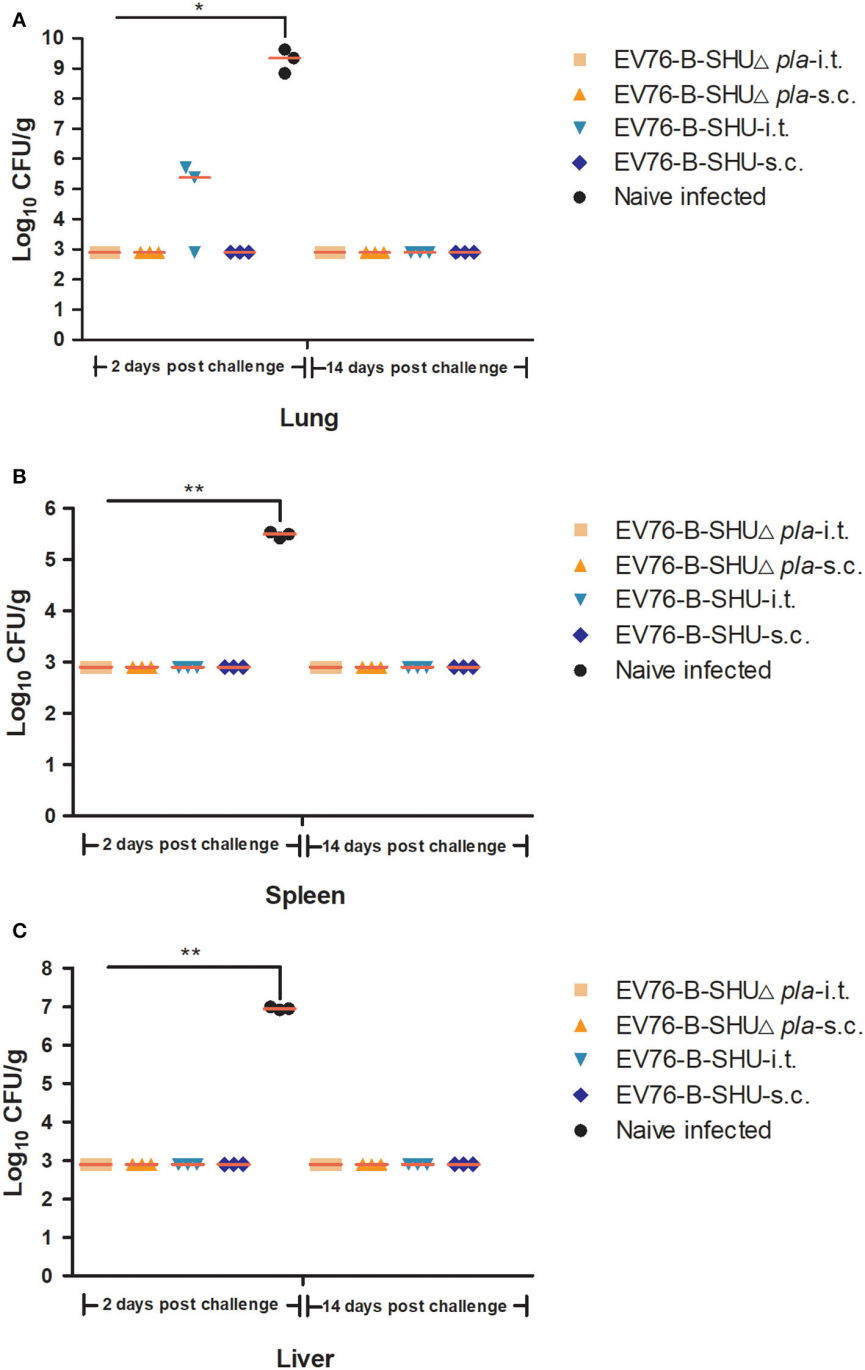
Bacterial loads in the organs of mice post-challenge. On days 2 and 14 after *Y. pestis* 201 challenge, 3 mice per group were sacrificed. Lungs, spleens, and livers were obtained to determine bacterial loads. **(A)** The bacterial loads in lungs of mice after challenge. **(B)** The bacterial loads in spleens of mice after challenge. **(C)** The bacterial loads in livers of mice after challenge. The experiments were performed twice independently with similar results. Data are expressed as the mean ± SD (*n* = 3) that collected from one representative experiment. *P < 0.05; **P < 0.01.

The *Y. pestis* loads in lungs, spleens and livers of mice were detected on day 20 after first immunization, day 20 after second immunization and day 20 after third immunization, respectively. As a result, no bacteria were detected in the lowest dilution of the samples, indicating that the immunized *Y. pestis* EV76-B-SHUΔ*pla* or EV76-B-SHU were presumably cleared from the mice at these time points. The mice were challenged with 61 LD_50_ *Y. pestis* 201 by i.t. on day 21 after the last immunization, and the bacterial loads in lungs, spleens, and livers of mice were determined on day 2 and day 14 post-challenge. On day 2 post-challenge, the bacterial counts in the lungs of EV76-B-SHUΔ*pla* and EV76-B-SHU immunized groups were markedly lower than that of the naïve mice control group (Figure 6A). Also, low levels of bacteria were detected in the spleens or livers of the EV76-B-SHUΔ*pla* and EV76-B-SHU immunized groups. In contrast, significant multiplication of *Y. pestis* 201 was observed in the spleens and livers of naïve mice (Figures 6B,C). On day 14 post-challenge, all naïve mice had succumbed to the *Y. pestis* infection, while low levels of bacteria were detected in the organs of EV76-B-SHUΔ*pla* and EV76-B-SHU immunized groups (Figures 6A–C).

### Histopathological Analysis of Mouse Tissues After Immunization and Challenge

**Figure 7:**
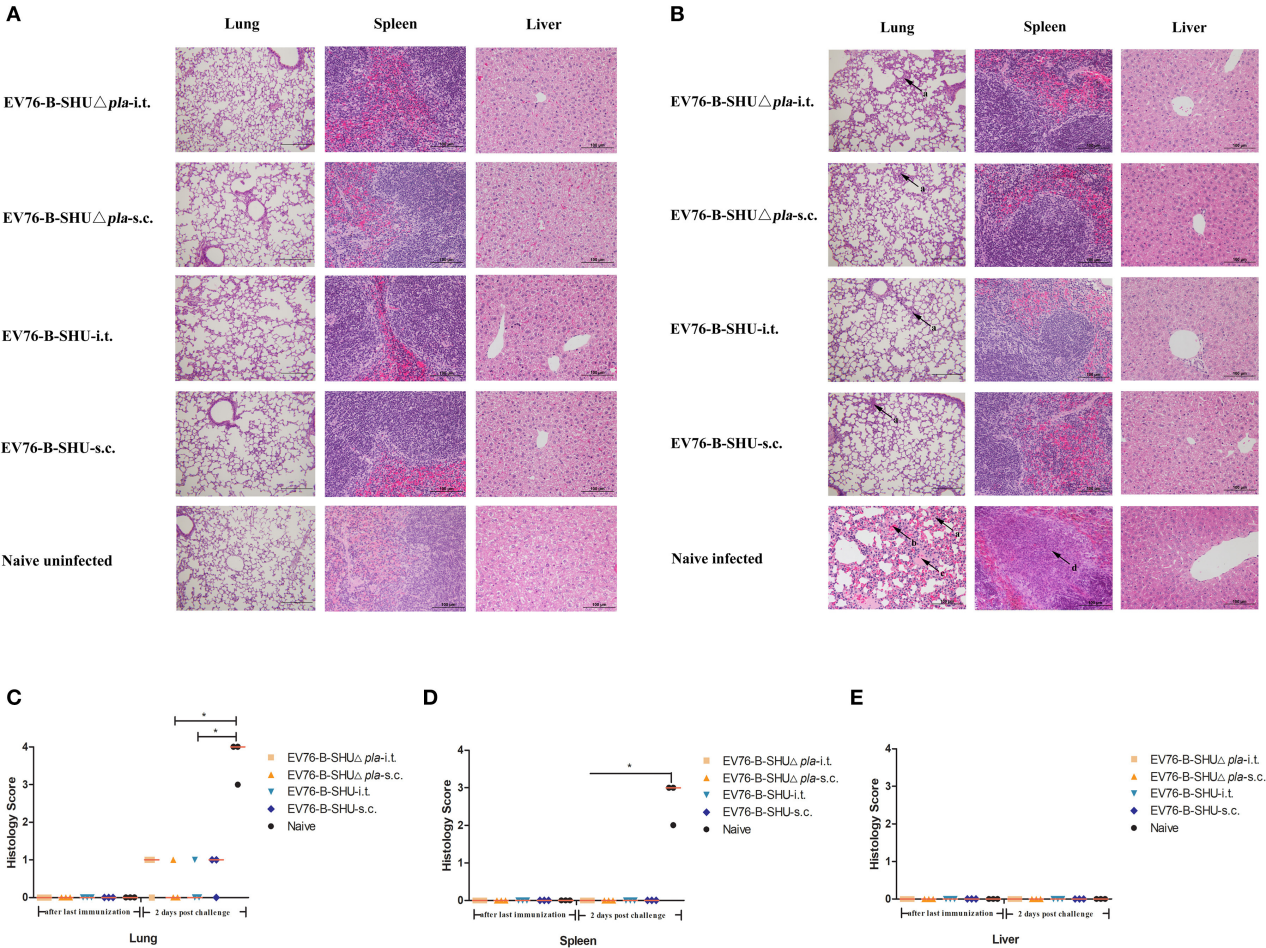
Pathological lesions in the tissues of mice after immunization and challenge. On day 21 after the last immunization and 2 days after *Y. pestis* 201 challenge, 3 mice per group were sacrificed. Lungs, spleens, and livers were harvested and fixed in formalin, embedded in paraffin, and stained with HE. The pathological changes were examined. **(A)** The pathological alterations in tissues after immunization. **(B)** The pathological alterations in tissues after challenge. **(C)** The pathological scores of lung tissue. **(D)** The pathological scores of spleen tissue. **(E)** The pathological scores of liver tissue. Tissue sections were evaluated by a trained pathologist according to the following scores: 0, no pathological lesions; 1, minimal; 2, mild; 3, moderate; 4, severe. The degree of pathological lesions was related to the distribution and severity of lesions as follows: (I) thickened alveolar walls; (II) edema; (III) tissue parenchymatous lesions, such as congestion and hemorrhage. Arrow a, thickened alveolar walls; arrow b, hemorrhage; arrow c, edema; arrow d, necrosis. The experiments were performed twice independently with similar results. Data are expressed as the mean ± SD (*n* = 3) that collected from one representative experiment. *P < 0.05.

On day 21 after the last immunization with EV76-B-SHUΔ*pla* or EV76-B-SHU and on day 2 post challenge, 3 mice per group were sacrificed; lungs, spleens, and livers were harvested; and the pathological alterations were examined. On day 21 after the last immunization, no pathological change was observed in the lungs, spleens and livers of mice immunized with EV76-B-SHUΔ*pla* or EV76-B-SHU via i.t. or s.c. or the naïve uninfected group (Figure 7A). There was also no significant difference in pathological scores between different groups (Figures 7C–E).

On day 2 post-challenge, naive-infected mice had severe thickened alveolar walls, edema and hemorrhage in the lungs, and the lung tissue structure was destroyed locally. Inflammatory necrosis was observed in the white pulp of the spleens of naïve-infected mice. No pathological change was found in the livers of naive-infected mice. In contrast, only minimal thickened alveolar walls was observed in the lungs of mice immunized with EV76-B-SHUΔ*pla* or EV76-B-SHU via i.t. or s.c., while no pathological change was observed in the spleens and livers of mice immunized with EV76-B-SHUΔ*pla* or EV76-B-SHU via i.t. or s.c. (Figure 7B). The pathological scores in the lungs of naïve-infected control group were significantly higher than EV76-B-SHUΔ*pla*-s.c. and EV76-B-SHU-i.t. groups (Figure 7C), while the pathological scores in the spleens of naïve-infected control group were significantly higher than EV76-B-SHUΔ*pla* immunized and EV76-B-SHU immunized groups, no significant difference between the EV76-B-SHUΔ*pla* immunized and the EV76-B-SHU immunized groups was observed (Figure 7D).

## Discussion

Due to the re-emergence of plague in many parts of the world and the discovery of antibiotic-resistant strains, it is necessary to develop effective vaccines against plague. The subunit plague vaccines based on F1 and/or LcrV are particularly effective in rodents and macaques (Agar et al., 2009; Qi et al., 2010; Quenee et al., 2011a,b), but they do not effectively protect African green monkeys against *Y. pestis*(Smiley, 2008b). Also, highly varied antibody titers were noticed during clinical trials of subunit plague vaccine (Quenee and Schneewind, 2009). Mice vaccinated with F1 antigen alone fail to protect against the infection of F1-negative *Y. pestis* (Friedlander et al., 1995). Polymorphism in the *Yersinia* LcrV antigen enables immune escape from the protection conferred by an LcrV-secreting *Lactococcus Lactis* in a pseudotuberculosis mouse model, indicating that an anti-LcrV-based vaccine should contain multiple LcrV clades if protection against the widest possible array of *Yersinia* strains is sought (Daniel et al., 2019). New live-attenuated plague vaccine candidates that stimulate comprehensive immune responses have shown promising results. The live-attenuated vaccine EV76, which has been widely used historically, can protect against pneumonic and bubonic plague, but it has many immune side effects, and its use is undesirable due to safety concerns (Meyer et al., 1974; Russell et al., 1995; Sun et al., 2011). The virulence of a *Y. pestis* Pgm^−^/Pla^−^ strain by the aerosol route was tested in monkeys in a previous study (Welkos et al., 2002). However, the safety and immunogenicity of the EV76 based Pla^−^ strain after administration via i.t have not yet been investigated.

To gain a better understanding of the safety and immunogenicity of EV76 based Pla^−^ live-attenuated strains, EV76-B-SHUΔ*pla* strain was constructed by deleting the *pla* gene of the live-attenuated vaccine EV76-B-SHU strain. Then the residual virulence and protective efficacy of the EV76-B-SHUΔ*pla* after i.t. and s.c. were further evaluated in a mouse model. The survival rates of EV76-B-SHUΔ*pla* groups were significantly higher than EV76-B-SHU groups after 10^4^ CFU or 10^6^ CFU infection via aerosolized i.t. In addition, mice infected with 10^8^ CFU EV76-B-SHUΔ*pla* by s.c. showed a significantly higher survival rate than mice infected with 10^8^ CFU EV76-B-SHU, indicating that the EV76-B-SHUΔ*pla* strain, which lacks both the *pla* gene and the *pgm* chromosomal fragment, has better immune safety. These results are consistent with those of a previous study, confirming that deletion of the *pla* gene reduces the *in vivo* virulence of *Y. pestis* (Welkos et al., 2002).

Previous studies have reported that *Y. pestis* live-attenuated vaccines can induce both humoral and cell-mediated immune responses (Tiner et al., 2016). The level of serum IgG titers induced by immunization with EV76-B-SHUΔ*pla* or EV76-B-SHU through i.t. were comparable to that induced by immunization with EV76-B-SHUΔ*pla* or EV76-B-SHU through s.c, demonstrating that i.t. immunization efficiently induces the specific humoral immune response. It has been reported that T cell-mediated immunity, including CD4^+^ and CD8^+^ immune responses, is critical for combating *Y. pestis*. For the CD4^+^ T cell immune response, Th1 immunity and Th2 immunity both play an important role in the clearance of intracellular *Y. pestis* (Parent et al., 2005; Philipovskiy and Smiley, 2007; Szaba et al., 2009). The Th1 immune response, in which cytokines like IFN-γ, TNF-α, and IL-2 play important roles, is necessary for plague prevention (Nakajima and Brubaker, 1993; Atreya et al., 2000; Brubaker, 2003; Elvin and Williamson, 2004; Nakayama et al., 2017). It was also reported that a live-attenuated Δ*yscB* mutant, which provides protection against bubonic and pneumonic plagues, induces elevated levels of IL-4 (Zhang et al., 2013). In our study, immunization with the EV76-B-SHUΔ*pla* through i.t. or s.c. induced comparable level of specific T cell responses to those induced by immunization with the EV76-B-SHU strain, indicating that deletion of *pla* or i.t. immunization didn't change the T-cell dependent immunogenicity of EV76-B-SHU.

The mucosal surface is a physical barrier that prevents microorganisms and foreign substances from entering the body. sIgA plays a crucial role in the mucosal immune response. The main functions of sIgA are the neutralization of toxins and the prevention of the invasion of pathogenic and commensal bacteria across the mucosal epithelial layer (Tripathi et al., 2006; Macpherson et al., 2008; Ali et al., 2013; Leong and Ding, 2014). On day 21 after the last immunization, the levels of sIgA against sonicated *Y. pestis* 201 in BALF of EV76-B-SHUΔ*pla*-i.t. and EV76-B-SHU-i.t. were significantly higher than those of sIgA levels in BALF of EV76-B-SHUΔ*pla*-s.c. and EV76-B-SHU-s.c., indicating that i.t. is superior to s.c. at inducing the antigen-specific mucosal immune response. These results also demonstrate that 3 immunization steps are necessary for inducing sIgA production in lung.

The protective efficacy of EV76-B-SHUΔ*pla* administered by i.t. and s.c. was evaluated in detail. After 3 vaccinations with EV76-B-SHUΔ*pla* or EV76-B-SHU and a lethal challenge of *Y. pestis* 201, 100% survival rates were achieved in all 4 immunized groups. On day 2 post-challenge, significantly lower bacterial loads were detected in the organs of mice in the EV76-B-SHUΔ*pla* immunized and the EV76-B-SHU immunized groups. The pathological lesions in the lungs, spleens, and livers of EV76-B-SHUΔ*pla* immunized mice were similar to those of the EV76-B-SHU immunized groups; both were markedly less severe than the pathological changes of mice in the non-immunized group. These results indicate that EV76-B-SHUΔ*pla*-i.t. immunization can reach a protective efficacy and bacterial clearance ability equivalent to EV76-B-SHUΔ*pla*-s.c., EV76-B-SHU-i.t., and EV76-B-SHU-s.c. immunization. However, the significantly higher level of sIgA antibodies induced by EV76-B-SHUΔ*pla*-i.t. or EV76-B-SHU-i.t. immunization didn't lead to a better protection, indicating that the role of specific sIgA in BALF to the protection against *Y. pestis* 201 need to be further investigated.

Mucosal immunity is generally considered to be effective at inducing both systemic and mucosal immune responses. Immunization via aerosolized i.t. can induce higher levels of mucosal antibodies, which play an important role in improving bacterial clearance and protective efficacy (Feng et al., 2019). Vaccination through this route overcomes the disadvantages of intranasal and intratracheal immunization, such as drug loss in the nose, throat, and upper airways, and invasive injury, making it a preferable immunization route. To the best of our knowledge, this is the first study where the aerosolized i.t. delivery of a vaccine is used to evaluate the protective efficacy of a *Y. pestis* live-attenuated vaccine in mouse model. Vaccination through this route can induce higher levels of mucosal antibodies, which play an important role in improving bacterial clearance and protective efficacy. The mechanisms by which the live-attenuated vaccine strain exerts immunoprotective effects after aerosolized i.t. delivery of a vaccine and the evaluation of its efficacy and immune safety in non-human primate models still require further research.

## Data Availability Statement

The datasets generated for this study are available on request to the corresponding author.

## Ethics Statement

The animal study was reviewed and approved by The Institute of Animal Care and Use Committee of the Academy of Military Medical Sciences.

## Author Contributions

LJ, DZ, JJ, and XX designed the experiments. JF, YD, XH, WL, ZL, and LD performed animal experiments. JF and YD finished other experiments together. JF, YD, and MF analyzed data and drew the figures. JF, HY, XZ, ZD, BW, JJ, and XX revised the manuscript. All authors approved the article.

## Conflict of Interest

The authors declare that the research was conducted in the absence of any commercial or financial relationships that could be construed as a potential conflict of interest.
