# Supplementary material for: Construction of a Live-Attenuated Vaccine Strain of Yersinia pestis EV76-B-SHUΔpla and Evaluation of Its Protection Efficacy in a Mouse Model by Aerosolized Intratracheal Inoculation
Source: Front Cell Infect Microbiol. 2020 Sep 8;10:473. doi: 10.3389/fcimb.2020.00473 (PMC7509399; doi:10.3389/fcimb.2020.00473)
Supplement: Supplementary file 1 [file Data_Sheet_1.PDF]

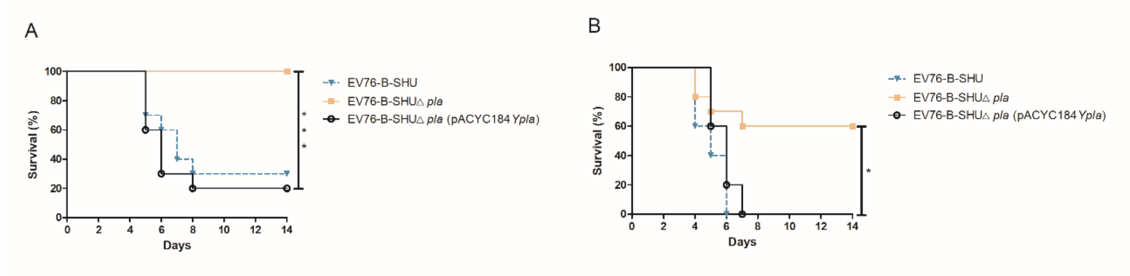

Fig S2. The complementation of virulence of EV76-B-SHU△*pla.*

(A) Ten mice per group were infected with dose of 10^6 CFU of EV76-B-SHU, EV76-B-SHU △*pla*, or EV76-B-SHU△*pla* (pACYC184*Ypla*) strains via i.t. route, respectively. (B) Ten mice per group were infected with dose of 10^8 CFU of EV76-B-SHU, EV76-B-SHU△*pla*, or EV76-B-SHU△*pla* (pACYC184*Ypla*) strains via s.c. route, respectively. Then mice were monitored for 14 days. Survival data were analyzed by using Kaplan–Meier survival estimates, and *P*-values of < 0.05 were considered statistically significant. Data are from one representative experiment of two independent experimental determinations.
