# Supplementary material for: Construction of a Live-Attenuated Vaccine Strain of Yersinia pestis EV76-B-SHUΔpla and Evaluation of Its Protection Efficacy in a Mouse Model by Aerosolized Intratracheal Inoculation
Source: Front Cell Infect Microbiol. 2020 Sep 8;10:473. doi: 10.3389/fcimb.2020.00473 (PMC7509399; doi:10.3389/fcimb.2020.00473)
Supplement: Supplementary file 2 [file Data_Sheet_2.PDF]

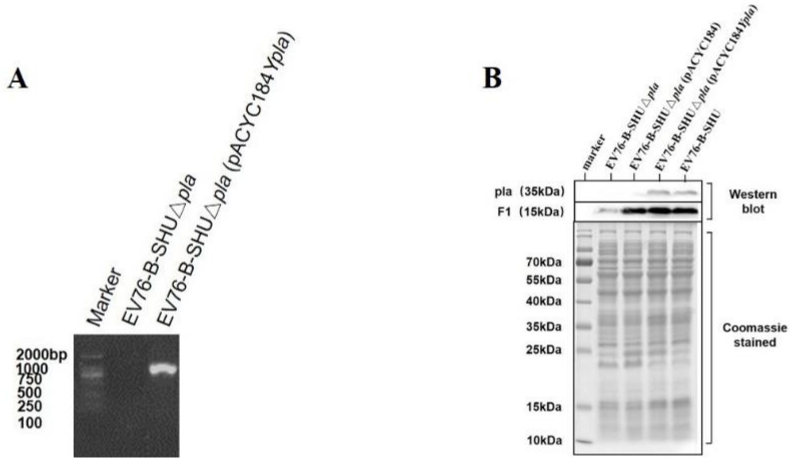

Fig. S1 Identification of *pla* gene in *Y. pestis* EV76-B-SHU△*pla* (pACYC184*Ypla*) strain and analysis of Pla expression in *Y. pestis* EV76-B-SHU△*pla* (pACYC184*Ypla*) strain.

(A) Identification of *pla* gene of *Y. pestis* EV76-B-SHU△*pla* (pACYC184*Ypla*) strain. (B) Analysis of Pla and F1 protein expression levels in *Y. pestis* EV76-B-SHU △ *pla* (pACYC184*Ypla*) strain by SDS-PAGE and immunoblot. The F1 protein levels in the EV76-B-SHU△*pla* mutant was less than that in other strains, which might be due to the transfer issue.
